# Small-volume point-of-care analytical methods

**DOI:** 10.1038/s41598-020-70903-4

**Published:** 2020-08-27

**Authors:** Chao-Min Cheng

**Affiliations:** Institute of Biomedical Engineering, National Tsing Hua University, Hsinchu, 300 Taiwan

**Keywords:** Blood urea nitrogen, Biochemical assays

## Abstract

Detecting clinically relevant diagnostic biomarkers, suitable for point-of-care detection, may facilitate rapid treatment and prevention of disease. However, medical diagnosis for which there are limited quantities of testable tissue or fluid, require point-of-care technologies with superior sensitivity and specificity. The purpose of this Editorial is to provide an overview of the Collection’s content, comprising original research into fluidics-based diagnostic platforms—specifically those made of paper or other low-cost materials, that can suitably operate with tiny sample volumes from mL to nL. In particular, we will focus on the clinical applications of such research, with potential uses in a number of fields including combating the COVID-19 pandemic for instance.

Designing diagnostic procedures, and improving their accuracy, has been a long-standing research focus that is constantly evolving as it incorporates recent pursuits and technologies from a range of areas, including analytical chemistry, bioanalytical chemistry, translational medicine, and medicine. With recent technological advances in multiple research fields—such as materials science, micro-/nano-technology, cellular and molecular biology, and bioengineering—attention is shifting toward the development of new diagnostic tools that not only address needs for high sensitivity and specificity, but that fulfill economic, environmental, and rapid point-of-care (POC) needs, for groups and individuals with constrained resources and, possibly, limited training.

Tang et al. refined the capacity of nuclear magnetic resonance relaxometry, successfully examining a tiny sample (below 20 μL), with a miniaturized NMR platform, at 150 °C and 900 bar pressure by integrating electronics, microfabrication, and novel chemistry^1^. Zhao et al. demonstrated a simple, universal approach for nucleic-acid-based pathogen diagnostics—requiring no electricity, or special equipment. The approach used a hand-powered syringe for the pump, with inexpensive amine-functionalized diatomaceous earth and a 1 μm Teflon filter as a reaction matrix in two stages of their process, which also used homobifunctional imidoesters. For a large sample volume (< 50 mL) they demonstrated a 100-fold better capture efficiency than that of a common commercial nucleic acid isolation kit^2^. Lode et al. reported on the development of a clinically based compounding approach, using silicone oil-free syringes and a 33 G × 9 mm low dead space hub injection needle. They have evaluated this approach for three anti-VEGF biologics commonly used in ophthalmology: aflibercept, ranibizumab (Lucentis), and bevacizumab (Avastin). Their results indicated that compounding and storage for 1 week did not compromise the functional activity of the biologics, and allowed for safe and cost-effective compounding of anti-VEGF biologics for intravitreal injections in prefilled silicone oil-free syringes^3^. Sypabekova et al. presented clinically relevant results regarding the detection of *Mycobacterium tuberculosis*-secreted MPT64 protein^4^. They used an interdigitated electrode as a platform to capture the immunogenic protein, and electrochemical impedance spectroscopy as a detection approach. Their assay involved a special receptor, a single stranded DNA aptamer that specifically recognized the MPT64 protein. An evaluation of this approach demonstrated 100% specificity for clinical serum and sputum samples, and a sensitivity of 88.24% and 76.47%, respectively^4^.

Microfluidic technologies, in particular, are considered very powerful tools for diagnosing and monitoring human diseases, and miniaturized fluidics-based platforms can precisely manipulate tiny body fluid volumes for rapid and accurate medical diagnoses. Yen et al. demonstrated a low-power microfluidic pump, based on travelling-wave electroosmosis, that could be operated at 1.5 V driving voltage, with a power consumption of 1.74 mW. This in-situ CMOS-based microfluidic pump is the first to drive a clinically diluted serum sample, driving a diluted (1:1,000 dilution ratio) serum sample with a flow rate of 51 μm/s^5^. Behrens et al. reported on a low-cost, open-source peristaltic pump, constructed using 3D-printed parts and common hardware that could be used with microfluidic devices for POC diagnostics. This pump accepted commonly available silicone rubber tubing in a range of sizes, from 1.5 to 3 mm, and was capable of producing flow rates up to 1.6 mL/min^6^. These microfluidic technologies may be used for a variety of POC diagnostic applications (e.g., infectious diseases, COVID-19 diagnosis) because they are disposable, inexpensive, portable, and easy to use, especially when they are manufactured using low-cost materials such as paper (and other materials such as cotton or bamboo^7,8^).

One of the purposes of this Collection in *Scientific Reports* is to bridge microfluidic technologies (polydimethylsiloxane-based and paper-based microfluidics) and biology with medicine, with a focus on applications of microfluidics for POC diagnostics. Tsai et al. have developed a multi-target lateral flow immunoassay strip, with a stacking pad design, to obtain on-site, rapid diagnosis of periprosthetic joint infection. The multi-target design of this device increases specificity, and the stacking pad enhances detection sensitivity^9^. Schwenke et al. developed a user-friendly lateral flow test that could detect very low amounts of free chlorine and demonstrated a 10 times higher sensitivity than a commercial dip test. This test could detect chlorine quantities as low as 0.05 ppm when oxidation stable flow test substrates were used, and results could be obtained within 4–5 min, with no additional action required by the operator^10^. Noh et al. developed a pipetting-based immunoassay using a removable magnetic ring-coupled pipette tip. Their “magnetic bead-capture antibody-targeted protein complex” was purified by pipetting and quantified by enzymatic color development, and could be integrated with a lateral flow system. This pipetting-based immunoassay was applied to detect the nucleoprotein (NP) of the influenza A virus. When this assay was applied exclusively for antigen capture in a lateral flow system, the limit of detection improved 100-fold and displayed greater sensitivity than the lateral flow system alone^11^. It is noted that this pipetting-based immunoassay may be used to detect SARS-CoV-2 infection. McArdle, H. et al. demonstrated an electrochemically based direct detection method using electrocatalytic platinum nanoparticles to detect 3 specific tRFs: 5′AlaTGC, 5′GlyGCC, and 5′GluCTC. Using synthetic tRF mimics, this system was linear over 9 orders of magnitude, with sub-attomolar limits of detection. Specificity was tested using naturally occurring mismatched tRF mimics. Further, McArdle et al. quantified tRF levels in patient plasma and showed that their detection system recapitulates results obtained by qPCR. They have also designed a tRF detection system with high sensitivity and specificity capable of quantifying tRFs in low plasma volumes using benchtop apparatus as well^12^.

The Collection has now been open for submissions since early 2019, and new submissions are still being welcomed. Here, we would like to show our deep appreciation to all authors and reviewers. Without their invaluable support and contributions, this Collection could not be published on schedule. This Collection may not cover all topics in this emerging scientific field—the development of practical tools for POC diagnostics. However, we firmly believe that our efforts would promote highly impactful innovations and challenging discussions in relevant academic and commercial communities.
